# Functional 20S Proteasomes in Retroviruses: Evidence in Favor

**DOI:** 10.3390/ijms252111710

**Published:** 2024-10-31

**Authors:** Vladimir Morozov, Alexey Morozov, Vadim L. Karpov

**Affiliations:** 1 Department of Infectious Diseases, Robert Koch Institute, 13353 Berlin, Germany; 2 Engelhardt Institute of Molecular Biology, Russian Academy of Sciences, Vavilov Street 32, 119991 Moscow, Russia; karpov@eimb.ru

**Keywords:** functional proteasomes, proteasome subunits, retroviruses, fractions, extracellular vesicles, viral cargo

## Abstract

Proteasomes are barrel-like cellular protein complexes responsible for the degradation of most intracellular proteins. Earlier, it has been shown that during assembly, hundreds of different cellular proteins are incorporated into retro-and herpes viruses. Among detected cellular proteins, there were different proteasome subunits (PS). Previous reports postulated the incorporation of 20S proteasome subunits and subunits of proteasome regulator complexes inside retroviruses. Here, we demonstrated the association of functional 20S proteasome with gammaretroviruses, betaretroviruses, and lentiviruses. Cleaved proteasome subunits β1, β2 and β5 were detected in tested viruses. Using fluorescent peptides and a cell-permeable proteasome activity probe, proteasome activity was detected in endogenous and exogenous retroviruses, including recombinant HIV-1. Taken together, our data favors the insertion of functional proteasomes into the retroviruses during assembly. The possible role of proteasomes in retroviruses is discussed.

## 1. Introduction

The Ubiquitin-proteasome system (UPS) degrades most intracellular proteins. The principal and minimal functional element of the UPS, responsible for the protein hydrolysis, is a 20S proteasome [1]. Each cell contains from 100K to 1M of proteasomes. The 20S proteasome is a 12–15 nm long barrel-like complex with a molecular weight of around 700 kDa and a half-life of about 1–5 days. It is composed of paired α(1-7) and β(1-7) subunits organized into four stacked heptametric rings. The α subunits form two external rings, while β-subunits form two rings between them. The α subunits maintain proteasome structure and prevent accidental substrate entry into its catalytic chamber, where β-subunits perform protein degradation [2]. Three of seven β-subunits demonstrate catalytic activities. The β1 subunit cleaves proteins after acidic amino acids, thus demonstrating caspase-like activity. The β2 subunit cleaves after basic amino acids, demonstrating trypsin-like activity, while β5 cleaves after hydrophobic residues, thus having chymotrypsin-like activity [3]. Importantly, catalytic beta subunits are integrated into the proteasomes containing propeptides that undergo autocatalytic cleavage at the last stage of proteasome assembly [4,5]. This ensures that the proteolytic activity is confined in the closed space of the inner chamber of the 20S proteasome. To degrade proteins marked for degradation with ubiquitin molecules, 20S particle associates with the multi-subunit 19S proteasome regulator, forming the 26S proteasome. The 19S regulator is composed of Rpt subunits 1-6 and Rpn subunits 1-5, 6-13 and 15. However, the 20S complex itself or in complexes with other regulators like PA28 or PA200 is capable of degrading peptides as well as certain proteins, including oxidized and damaged substrates [6,7,8]. Proteasome-generated peptide fragments about 3 to 22–24 amino acids in length [9], with several exceptions, are further degraded by cytosolic peptidases [10,11].

Retroviridae is a large family of enveloped RNA viruses that contain two identical single-stranded copies of genomic RNA. The genomic RNA (gRNA) of retroviruses is converted to viral DNA (vDNA), which is transported to the cellular nucleus and integrated into the host genome. The Retroviridae family is divided into two subfamilies (i.e., Orthoretroviruses and Spumaretroviruses). There are six orthoretrovirus genera: alpha-, beta-, gamma-, delta-, epsilonretroviruses, and lentiviruses. Assembly of the hexametric Gag lattice of alpha- and gammaretroviruses occurs at the cell membrane, while capsid assembly of beta-retroviruses takes place in the cytoplasm. Release of assembled retroviruses occurs by budding using the cellular membrane as an envelope. The process of budding by itself is rather complex and requires “help” from several host factors like ESCRT [12]. During the assembly and budding of retroviruses, diverse intracellular elements (“cargo”), such as peptides, proteins, hormones, RNA, and DNA fragments, can be trapped within the viral particles.

Previously, several reports postulated the presence of cellular cargo in lentiviruses that were released from the epithelial cells and lymphocytes, reviewed in [13]. Later, Chertova et al. examined the cargo of lentiviruses from monocyte-derived macrophages in more detail [14]. In particular, proteasome subunits were detected among cellular elements [14,15,16,17,18,19]. Thus, the α subunit of the PA28 proteasome regulator, Rpn 1 and Rpn 11 subunits of the 19S regulator were among 235 identified cellular proteins [14]. Santos et al. revealed 19S subunits Rpt 5, Rpn 3, 6, 8, and one 20S β6 subunit in HIV-1 virions [20]. Brégnard and colleagues identified 19S subunits Rpt 1, Rpt 6, Rpn subunits 3, 6, 7, and again 20S β6 subunits within the HIV-1 particles [21]. Furthermore, Stephenson and colleagues [18] detected in SIV-1 from sooty mangabeys and rhesus macaque’s 20S proteasome α1, α4-7, β1-6 subunits, as well as the 19S Rpn subunits 1, 6, and 11. In either of the investigated samples, additional subunits were revealed: α3 and α8, β7-10, 19S subunits Rpt 2-5, Rpn subunits 2, 3, 5, 8, 9, as well as PA28 α and β subunits [18]. Thus, almost all the 26S proteasome subunits were detected in viral particles. However, most proteasome subunits were revealed using mass-spectrometry and the presence of functional proteasomes in retroviruses was not previously investigated. Here, we provide arguments in favor of functional proteasomes in retroviruses.

## 2. Results

### 2.1. Parameters and Buoyant Density of Free 20S Proteasomes

**Figure 1:**
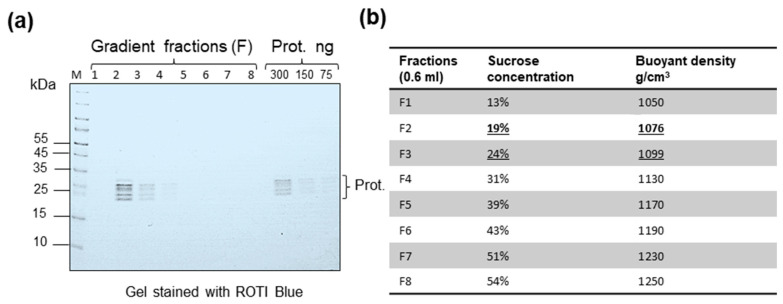
Estimation of buoyant density of 20S proteasomes after sucrose density gradient centrifugation. (**a**) One μg of commercial 20S proteasome was analyzed by sucrose (15–55%) density gradient centrifugation. After 5 h of high-speed centrifugation, eight fractions (F) were collected, pelleted, and analyzed by gradient SDS-PAGE. The gel was stained with ROTI Blue quick (Carl Roth, Karlsruhe, Germany). Tracks: 1–8—gradient fractions (F). The proteasome subunits were detected predominantly in F2 (1076 g/cm^3^) and significantly less in F3 (1099 g/cm^3^). Titration of commercial 20S proteasome (Prot.): 300 ng, 150 ng, 75 ng is shown. (**b**) The buoyant density of fractions and density of proteasome subunits (underlined).

Proteasomes are among the smallest structural elements in cells. They are minimally composed of 28 protein subunits. Extracellular circulating 20S proteasomes were previously detected in animals (including fetal calf serum) and humans [22,23,24]. As a result, proteasome “contamination” of FBS could be anticipated. Therefore, the initial part of the study was focused on the proteasome parameters. We estimated the buoyant density of 20S core particles using as a reference highly purified commercial 20S proteasomes. One μg of the commercial sample was diluted in 500 μL of TN buffer and was loaded on a 15–60% sucrose density gradient. After ultracentrifugation, eight fractions (F) were collected, and buoyant density was estimated; samples were pelleted and dissolved in PBS to obtain samples concentrated 250 times. The samples were examined using gradient SDS-PAGE followed by gel staining with ROTI Blue quick dye. In our hands, the protein detection limit was 25 ng–50 ng per band. The revealed protein pattern included three discrete protein bands and two protein pairs (proteins with close MW underlined): 25.7; 26.4; 27.3; 27.8; 28.3; 29.5; 29.8 kDa. It was shown that proteasome subunits located predominantly in F2 (buoyant density 1076 g/cm^3^) (Figure 1a). Only traces of proteasome subunits were present in F3 (buoyant density 1099 g/cm^3^) (Figure 1a).

Based on these data, we concluded that a high-speed gradient centrifugation allows to separate circulating proteasomes (below buoyant density 1.10 g/cm^3^) from circulating retroviruses (buoyant density 1.17–1.18 g/cm^3^).

### 2.2. Extracellular Vesicles (EV) in Cell Supernatant

There are several issues that need to be solved in order to obtain pure retroviruses for the proteasome study. The problem concerns extracellular vesicles (EV) released from cells. As the EVs are released from the same cells during virus processing, they “contaminate” pool of retroviruses and complicate purification.

**Figure 2:**
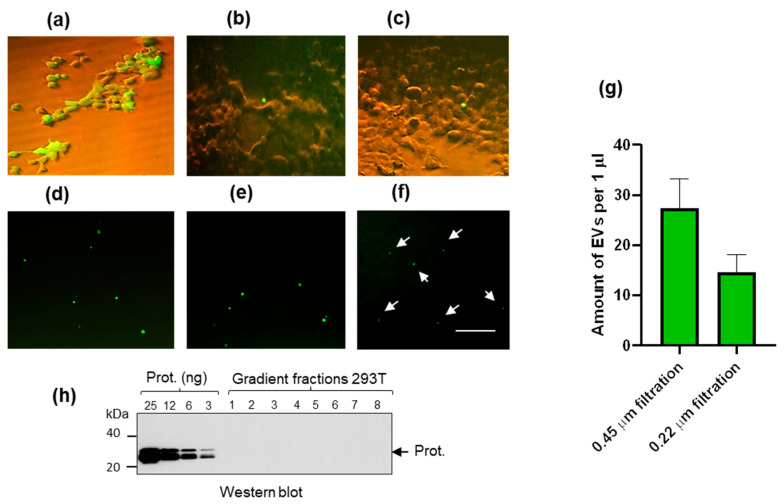
Release of presumed MVs from HEK293T cells expressing EGFP and quantification of fluorescent vesicles. (**a**) Expression of EGFP in HEK293T cells after 16 h in culture. (**b**,**c**) “Pre-detachment” stage of large MVs. (**d**,**e**) Floating extracellular vesicles in cell-free supernatant after filtration through the 0.45 µm and (**f**) through the 0.22 µm membranes, respectively. Bar = 100 µm. Arrows indicate fluorescent particles. (**g**) Estimation of the amount of EVs in 1 µL of cell culture media by flow cytometry. Estimations were performed in triplicates (**h**). Sucrose gradient centrifugation of cell supernatant from HEK293T cells. Each fraction was concentrated 250 times. No proteasome subunits were detected in analyzed gradient fractions. Western blotting was performed using mouse anti-20S alpha 1, 2, 3, 5, 6, 7 proteasome subunits. The limit of the detection by Western blot assay was ~1 ng/band.

To estimate amounts of budding EVs, we used HEK293T cells that express an enhanced green fluorescent protein (EGFP). To prevent contamination with vesicles from FCS, we substituted the FCS-containing medium with the FCS-free medium. Sixteen hours post-splitting expression of EGFP in cells was detected (Figure 2a). Quantification of fluorescent EVs in cell supernatant was performed after 72 h (Figure 2b,c). Cell supernatant was twice centrifuged at a low speed and filtered. Half of the supernatant was filtered through the 0.45 μm membrane (Figure 2d,e), and the other half was filtered through the 0.22 μm membrane (Figure 2f), respectively.

After filtration through the 0.45 µm membrane, there were about 8–10 vesicles in the field (Figure 2d,e), and after filtration through the 0.22 µm membrane, about 4–6 vesicles were detectable (Figure 2f). It could not be ruled out that some fluorescent spots may represent bright GFP aggregates. To this end, additionally, obtained supernatants were examined by flow cytometry since particle fluorescence has no effect on the forward scatter (FSC) that is proportional to the diameter of the particle. The detection limit of flow cytometry is around 150–200 nm. As a result, on average, 26 and 13 particles per microliter of media were identified following filtration through 0.45 and 0.22 µm membranes, respectively (Figure 2g). However, considering the detection limit of the flow cytometry, it might be expected that the actual number of vesicles in media is presumably more significant. Together, obtained data demonstrate that vesicles were not numerous, filtration through the 0.22 µm membranes reduced the number of “large” vesicles (>250 nm), and as expected, 2–3 times smaller particles were registered (Figure 2f).

Next, we attempt to detect 20S proteasomes in supernatant from HEK293T cells as described in Materials and Methods (Figure 2h). In brief, after centrifugation followed by sucrose gradient centrifugation, fractions were collected; pellets were recovered, diluted, and concentrated 250 times. In the obtained fractions, no α subunits of 20S proteasomes were detected by Western blot, indicating that the amount of proteasomes in cell supernatant was below our detection limit of 1–2 ng/band (Figure 2h).

### 2.3. Attenuated Variants of HIV-1 for the Proteasome Studies

**Figure 3:**
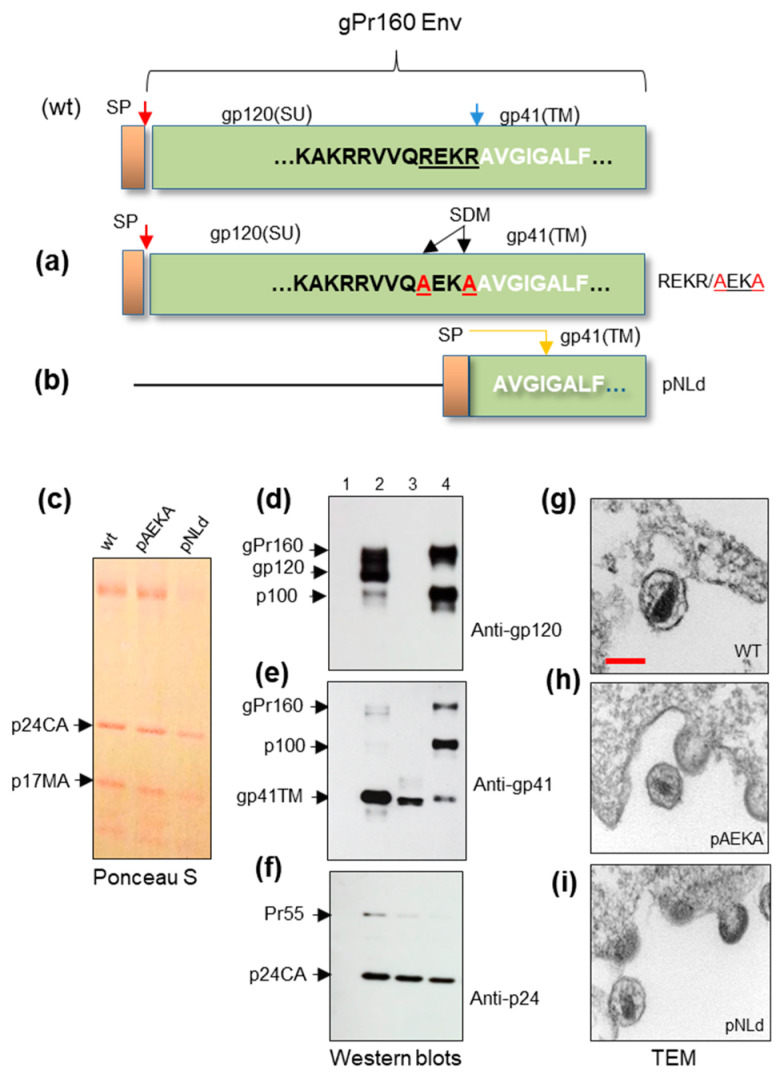
The HIV-1_pNL4-3_ with modifications in the envelope (*env*) gene. Protein pattern and morphology of particles. (**a**) By SDM, the C-terminal part of SU (REKR) was changed for the “AEKA”. (**b**) pNL4-3 with truncated SU and signal peptide (SP) ligated to TM. (**c**) Protein pattern of virus-like particle after transfer and staining of the membrane. (**d**–**f**) Western blot analyses of modified viruses using anti-gp120 SU, anti-gp41 TM and anti-p24 CA sera, respectively. Track 1—pelleted supernatant from non-infected HEK293T cells (negative control). Track 2—wild type HIV-1_pNL4-3_. Track 3—pNLd. Track 4—pNLAEKA. (**g**–**i**) TEM of modified HIV-1 released from transfected HEK293T cells. Morphology of HIV-1 wt, HIV-1 without SU-TM cleavage site (AEKA) and HIV-1 without SU are shown. The blue arrow marks the SU-TM cleavage site. The yellow arrow indicated the presumed SP cleavage site. The black arrow marks two (underlined) substitutions in the SU-TM cleavage site. Red bar = 100 nm. Ultrathin sections of samples were examined using a JEM-2100 electron microscope (JEOL Csorp., Akishima, Japan) operated at 200 kV. Images were recorded with a CCD camera and 2048 × 2048 pixels. Pellated supernatants were dissolved in PBS.

To prevent contamination and avoid infection, the envelope of the infectious molecular clone HIV-1_pNL4-3_ was modified. The first modification concerns the SU-TM cleavage site. By site-directed mutagenesis (SDM), the SU-TM furin cleavage site was changed at the 3′-end of the SU (Figure 3). The “REKR” motif (3′-env gene) of the plasmid was modified for the “AEKA”motiv (named pNLAEKA) (Figure 3a). Another modification was SU truncation, and a signal peptide sequence was ligated to TM (named pNLd). After the transfection of cells released, recombinant viruses were purified on a sucrose gradient, and pellets were examined by Western blotting (Figure 3d–f). Thin-section and electron microscopy were performed on virus-producing cells (Figure 3g–i).

As expected, modified (REKR/AEKA) viruses contained a significant amount of unprocessed gPr160 Env and non-glycosylated p100. A low amount of SU glycoprotein was detected, while pNLd demonstrated the absence of env proteins (Figure 3d). The presence of a low amount of SU in pNLAEKA samples might be explained by some furin activity against the presumed secondary target. At the same time, the morphology of viral capsids was not changed.

### 2.4. Comparison of Purification Procedures

The pureness of retroviruses is crucial for the analyses of virus-associated proteasomes. To optimize virus purification, we compared two purification procedures using supernatant from HEK293T cells expressing porcine endogenous retroviruses (PERV). Both purification techniques include low-speed centrifugation, filtration, and high-speed centrifugation through the sucrose cushion. After two rounds of low-speed centrifugation, half of the supernatant was filtered through the 0.45 µm membrane, followed by high-speed centrifugation through the 20% sucrose cushion. Remained supernatant was filtered through the 0.22 µm membrane, followed by high-speed centrifugation through the 25% sucrose cushion. The obtained pellets were dissolved in TN buffer to gain 250 times the concentration.

**Figure 4:**
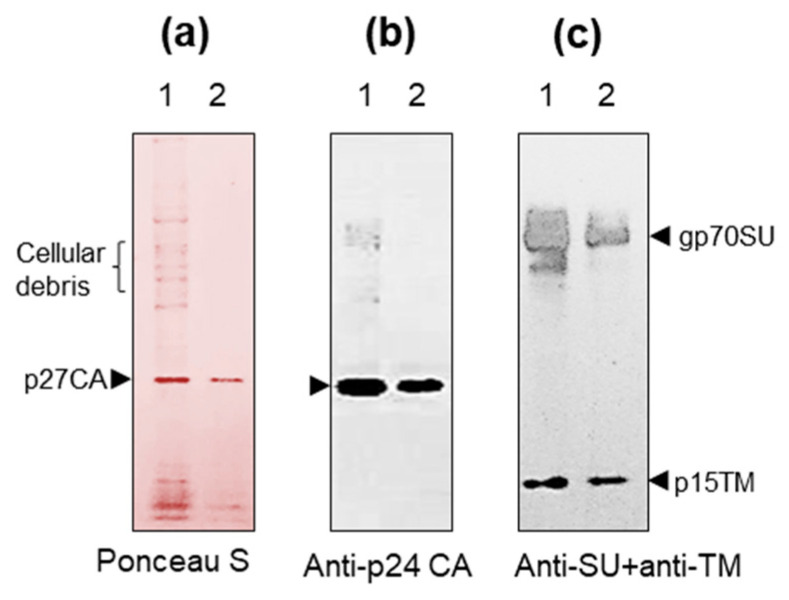
Two procedures for retroviruses purification (PERV). (**a**) Viral proteins after SDS-PAGE and protein transfer on the nitrocellulose membrane. The membrane was stained with Ponceau S. After destaining, the nitrocellulose membrane was used for the Western blot analyses. Track 1—Filtration of cell supernatant through the 0.45 μm filter followed by centrifugation through the 20% sucrose cushion. Track 2—Filtration of cell supernatant through the 0.22 μm filter followed by centrifugation through the 25% sucrose cushion. In each case, PERV was concentrated 250 times. (**b**) Comparative Western blot analysis of samples using anti-p27 CA and (**c**) comparative Western blotting of samples using anti-surface (SU) glycoprotein and anti-transmembrane (TM) protein sera together.

After SDS-PAGE and protein transfer, the nitrocellulose membrane was stained with Ponceau S to confirm the quality of transfer (Figure 4a). Western blot analyses were performed using anti-p27CA (Figure 4b), and after stripping, the membrane was treated with mixed anti-gp70SU and anti-p15TM sera (Figure 4c). For further experiments, we used the second procedure of purification, as the number of cellular-specific proteins was significantly reduced, even though the amount of virus-specific protein was diminished (Figure 4).

### 2.5. Analyses of Purified Viruses and Quantification of the CA Content

After initial centrifugation steps, membrane filtration and centrifugation through the sucrose cushion, viruses were purified by sucrose density gradient centrifugation. To increase the number of particular viruses, several of them were prepared two or three times.

**Figure 5:**
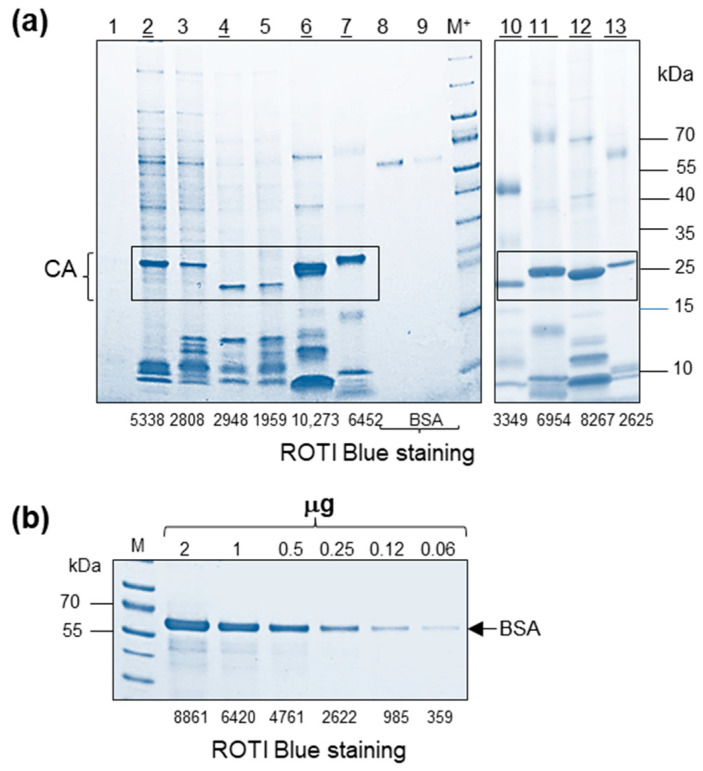
Protein pattern of retroviruses after sucrose gradient centrifugation. Estimation of the CA contents. (**a**) Examined fractions with buoyant density 1.15 g/cm^3^–1.17 g/cm^3^. Track 1—HEK293T (negative control); Tracks 2,3—exogenous JSRV load 7 μL; Track 4—pNLAEKA load 7 μL; Track 5-pNLd load 7 μL; Track 6—endogenous FeLV load 1 μL; Track 7—endogenous RD-114 load 1 μL; Track 8-BSA 250 ng; Track 9—BSA 125 ng; Track 10—exogenous MMTV load 5 μL; Track 11—endogenous RD-114 load 1 μL; Track 12—endogenous FeLV load 1 μL; Track 13—endogenous PERV load 7 μL. CA proteins are given in a frame. (**b**) Serial two-fold dilutions of bovine serum albumin (BSA) were used as a reference for the CA of retroviruses. Centrifugation was performed for 2 h at 110,000 rpm. Numbers below the figures (**a**,**b**) represented relative band intensity estimated using ImageJ software. Viruses (tracks 2, 4, 6, 7, 10, 11, 12, 13) selected for further proteasome analyses are underlined. Gels were stained with ROTI Blue quick (Carl Roth, Karlsruhe, Germany). M+-color gel markers mixed with ECL markers.

Here, we analyzed three endogenous retroviruses: PERV, RD-114, and FeLV, and two exogenous retroviruses: JSRV and MMTV. We also tested virus-like particles produced in HEK293T cells transfected with modified expression vectors (pNLAEKA, pNLd) based on HIV-1_pNL4-3_ (Figure 5). The following viruses, PERV, pNLAEKA, pNLd, and JSRV, were purified using initially the second established procedure (Figure 4), followed by sucrose gradient centrifugations. The quality of the samples was good, and only minimal background was observed in some of the samples (Figure 5a). Pelleted fractions were examined by electrophoresis in SDS-PAGE gradient gel, followed by gel staining with ROTI Blue quick. Aliquots from selected fractions (buoyant density 1.15–1.17 g/mL) were analyzed using 4–20% gradient SDS-PAGE, followed by gel staining. In addition to the above-mentioned purified viruses, double sucrose density gradient preparations of FeLV and RD-114 were examined. Depending on retrovirus genera, processing of Gag precursor results in three to six proteins in equimolar amounts. Thus, the profiles of low molecular mass proteins in retroviruses were different. Viral preparations with non-specific protein debris were not used in further analyses.The CA content was quantified using the ImageJ ver. 1.53o software (Figure 5b), revealing about five times the difference between examined viruses (Figure 5a). The obtained results were confirmed by serial dilutions of BSA (Figure 5b). In this regard, for further experiments, concentrated samples were diluted accordingly.

It is worth mentioning that the sensitivity of the protein staining depends on the protein load, parameters of the gradient gel and dyes used for staining. Estimation of CA band intensities revealed 0.97 µg in 7 µL load in JSRV (F4) and 0.4 µg in 7 µL of pNLAEKA (F4). About 1.24 µg of CA were detected in 1 µL of RD-114 and 1.67 µg in 1 µL of CA of FeLV, respectively. Estimation of all the CA content in retroviruses is summarized (Table 1).

Quantification of the CA in retroviruses allows proper adjusting of retrovirus amount and further estimation of proteasome content.

### 2.6. Proteasome Subunits Revealed in Retroviruses

**Figure 6:**
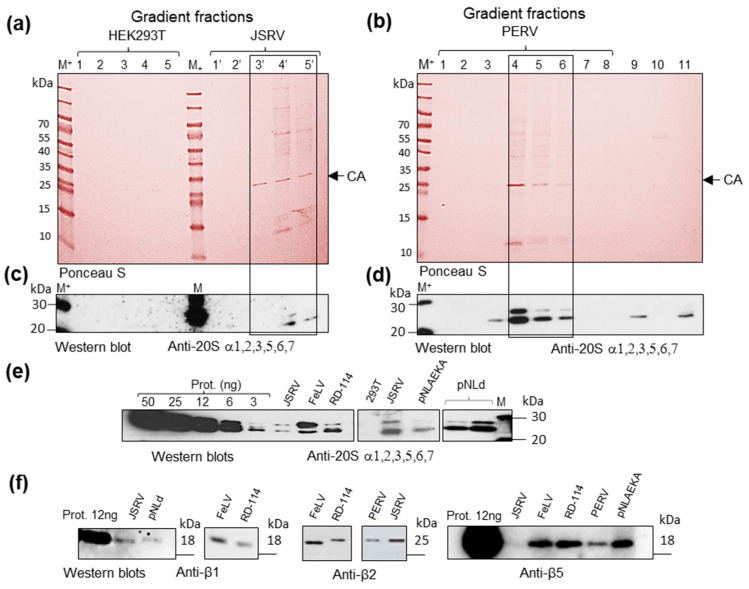
Proteasome α and β subunits were detected in sucrose gradient fractions of JSRV, PERV, FeLV, RD-114, and recombinant HIV-1 (pNLd and pNLAEKA). (**a**) Nitrocellulose membrane after SDS-PAGE, protein transfer and Ponceau S staining. The membrane contains gradient fractions F1–F5 of the supernatant from HEK293T cells and fractions F1′–F5′ obtained from supernatant of JSRV-producing cells. CA (arrow) indicates JSRV capsid proteins. Numbers above the image indicate fraction numbers. (**b**) Nitrocellulose membrane containing gradient fractions F1–F8 from the supernatant of PERV-producing cells stained with Ponceau S. CA (arrow) indicates PERV capsid proteins. Numbers above the image indicate fraction numbers. Track 9: PERV gradient fraction F3 grown on FCS-free medium. Track 10—gradient fraction F4 obtained from the supernatant of non-infected HEK293T cells (control). Track 11—20S proteasome 3 ng (control). (**c**) Western blot analysis of gradient fractions F1–F5 from HEK293T cells and gradient fractions F1′–F5′ from JSRV-producingcells with antibodies to 20S proteasome α 1, 2, 3, 5, 6, 7 subunits. (**d**) Western blot analysis of proteasome control and gradient fractions obtained from PERV-producing cells with antibodies to 20S proteasome α 1, 2, 3, 5, 6, 7 subunits. (**e**) Serial dilution (from 50 ng to 3 ng) of commercial 20S proteasome (Enzo, Farmingdale, NY, USA) and detection of proteasome alpha subunits in retroviruses from gradients fractions (F4, F5) using anti-20S proteasome α 1, 2, 3, 5, 6, 7 serum. (**f**) Detection of proteasome beta subunits in fractions of gradient purified retroviruses by Western blotting using anti-β1, anti-β2, and anti-β5 antibodies. Isolated viruses were from fractions F4 and F5. The 12 ng of commercial 20S proteasome (Enzo, Farmingdale, NY, USA) was used as the positive control. M+-Size markers mixed with ECL-size markers. Positions of marker proteins are indicated with bars.

Initially, we analyzed if viral preparations contain 20S proteasome α-subunits. These subunits were detected in all viral samples (Figure 6a–e). To determine if viral particles contain assembled proteasomes, we investigated the presence of proteasome β-subunits with cleaved pro-peptides in viral preparations. Since cleavage of β subunit precursors happens at the latest stages of proteasome assembly, cleaved subunits would indicate the existence of complexes. Cleaved proteasome subunits could be distinguished from precursors due to a lower molecular weight, and only cleaved subunits are found in commercial preparations of 20S proteasomes. Western blot analysis of JSRV (F4), PERV (F4), and pNLAEKA (F4), as well as of FeLV and RD-114 samples, revealed cleaved proteasome β1, 2 and 5 subunits with molecular weight that corresponds to β-subunits found in commercial proteasome preparations (Figure 6f). These results indicate the association of assembled 20S proteasomes with viral particles.

### 2.7. Functionality of Virus-Associated Proteasomes

**Figure 7:**
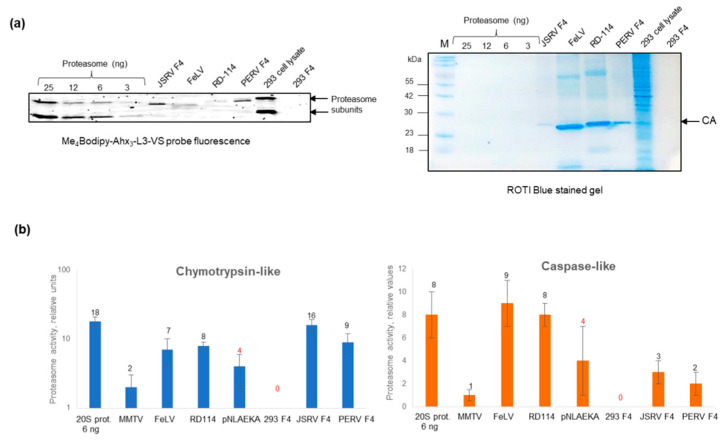
Analyses of proteasome activity using Me_4_Bodipy-Ahx_3_-L3-VS cell-permeable proteasome activity probe and fluorogenic peptides. (**a**) Samples were incubated for 1 h with the probe and loaded into the 15% Tris-glycine polyacrylamide gel. The gel was analyzed at the excitation wavelength 480 nm and emission wavelength 530 nm (**left panel**). Fifteen microliters of gradient fractions were loaded. Two microliters of diluted FeLV and RD-114 virus preparations were used. The same gel was stained using ROTI Blue quick (Carl Roth, Karlsruhe, Germany) protein stain (**right panel**). M-Prestained Protein Marker II (10–200 kDa) (Servicebio, Wuhan, China). (**b**) Chymotrypsin-like and caspase-like proteasome activities in viral preparations and gradient fractions. Fractions (F4) of gradient-purified supernatant from non-infected HEK293T cells and HEK293T cells transfected with JSRV, PERV, and pNLAEKA expression clones were used. Chymotrypsin-like and caspase-like proteasome activities were determined using fluorogenic substrates Suc-LLVY-AMC and Z-LLE-AMC, correspondingly. Six microliters of gradient fractions were used. Two microliters of diluted FeLV and one microliter of RD-114 virus preparations were used. Only five microliters of MMTV were used as the amount of sample was limited. Tests were performed in triplicates. The experiments with MMTV were performed in duplicates.

To determine the functionality of viral-associated proteasomes, we used cell-permeable proteasome activity probe Me_4_BodipyFL-Ahx_3_Leu3VS. Analysis of gels containing samples following the incubation of virus preparations with the probe revealed that JSRV (F4) and PERV (F4) contain active proteasome subunits with activity comparable to 3–6 ng of purified human 20S proteasome (Figure 7a). In the case of FeLV and RD-114, the activity was also detected, although revealed bands (FeLV) were positioned differently. No signal was seen in HEK293T (F4) (Figure 7a).

To confirm the presence of functional proteasomes in virus preparations, we estimated chymotrypsin-like and caspase-like proteasome activities using fluorescent peptides (Figure 7b). Valuable chymotrypsin-like activity was revealed in JSRV and PERV gradient fractions F4, RD-114 and FeLV. The activity in JSRV (F4) was comparable to the activity of the 6 ng of purified human 20S proteasome. Certain activity was also observed in pNLAEKA (F4). The strongest caspase-like activity was detected in FeLV and RD-114 (F4). Obtained results confirm the association of functional proteasomes with viral particles.

## 3. Discussion

Here, we aimed at the determination of active proteasomes in purified betta- and gammaretroviruses and recombinant lentiviruses. Previously, 26S proteasome subunits were identified by mass-spectrometry inside different viruses [14,17,18]. However, the presence of functional proteasomes in virus particles was not addressed. We have shown that highly pure preparations of different retroviruses contain functional 20S proteasomes. Thus, we obtained evidence in favor of proteasome integration into retroviral particles. 

We sought to estimate the putative number of proteasomes in viruses. To begin with, we attempt to number proteasomes that theoretically might be present inside the virion. Indeed, the size of retroviral virion, on average, is ~100–120 nm in diameter. Consequently, the volume of “empty virus” is about 523,250 nm^3^. The dimension of 20S proteasomes is 12–15 nm, and hence the volume is about 1696 nm^3^. Thus, theoretically, 308 of the 20S proteasomes can potentially accommodate the space of a single virus. At the same time, the volume of viral capsid, on average, is 65,125 nm^3^. If not counting gRNA, free Gag and cellular proteins in the capsid, the maximum amount of 20S proteasomes may be less than 38 inside and ~270 outside of the capsid. Evidently, considering numerous free viral and non-viral proteins inside virions, there might be fewer spaces for the proteasomes. It is considered that a single virion contains 0.1 fg of CA proteins or ~1500–2000 molecules [25]; thus, 1 ng of capsid protein is equal to 10^7^ capsids. Considering our estimations (Figure 5 and Figure 7, Table 1), one microliter of JSRV (F4) contains 1.39 × 10^9^, pNLAEKA (F4) ~5.7 × 10^8^ of capsids, respectively. While one microliter of FeLV contains 1.4 × 10^10^ and RD-114 ~8.4 × 10^9^ capsids (Table 1).

Using ImageJ software, it was determined that in 6 µL of JSRV (F4), the amount of proteasome α-subunits is comparable with the amount of proteasome subunits detected between 3 ng and 6 ng of commercial 20S proteasome (Figure 6). Accordingly, 6 µL of pNLAEKA (F4) contains less than 1 ng of α-subunits; one microliter of FeLV—the number of subunits comparable to 6 ng of purified proteasomes, while one microliter of RD-114—of 3 ng of purified proteasomes (Figure 6). However, we cannot exclude that some proteasome α-subunits associated with viral particles could be present as individual subunits which are not integrated into proteasomes. Therefore, calculations based on α-subunit presence might not be entirely correct. Measurements of proteasome activity reveal chymotrypsin-like activity in 6 µL of JSRV gradient fraction comparable with the activity of 6 ng of purified 20S proteasomes for 2 µL of FeLV and 1 µL of RD-114—3 ng of proteasome (Figure 7). Using a proteasome activity probe, we revealed that 15 µL of JSRV (F4) and 15 µL of PERV contain the amount of active proteasome subunits higher than in 3 ng but less than in 6 ng of purified proteasomes (Figure 7). Considering that 1 ng of 20S proteasomes is equal to 10^9^ proteasome particles for JSRV F4, we roughly obtain around 0.2–0.3 proteasome per one capsid and 0.5–1 proteasome per the PERV (F4) capsid. Further on, for FeLV, we obtain 0.1 and for RD-114—0.12 proteasomes per capsid, respectively. 

It should be emphasized that we cannot entirely exclude “contamination” of viral preps with extracellular vesicles [26,27]. The membrane-encapsulated EVs are variable and numerous [28]. They are defined by physical parameters, function, composition and genesis. Among characterized subtypes are exosomes (~100 nm in diameter), apoptotic bodies (about 1–5 μm in diameter) and microvesicles (MVs, 100–1000 nm in diameter) [29]. Exosomes are small and can traverse cell barriers, including the blood-brain barrier. The apoptotic bodies are large and frequently have amorphous structures that form during cell death. MVs, as retroviruses, bud off the membrane and thus trap membrane fragments containing complexes of glycoproteins, phospholipids and receptor ligands. Moreover, MVs contain micro-RNA, proteins of cytoplasmic origin, and, importantly, include proteasomes [30,31,32]. MVs cargo might be different depending on the cell type and condition [33]. Recently, we have shown that during assembly and budding, MVs can highjack not only elements of cells but also assembled retroviruses [34]. It should be highlighted that cellular cargo delivered by MVs is not a “passive garbage”, conversely, individual elements of cargo can be involved in immune regulation, cell signaling and other processes [28]. Since MVs are variable in size and buoyant density. Theoretically, a certain amount of them might have parameters matching both—the diameter and buoyant density of retroviruses. At the same time, in contrast with MV variability, the parameters of retroviruses are mostly “identical”. Each mature retroviral particle contains about 1500–2000 capsid (CA) molecules that assemble into the viral capsid [25]. Interestingly, the gRNAs are also participating in the formation of the viral capsid [35]. Thus, the assembled virion has a regular structure, including gRNA as a stable “heavy element”. In this regard, during sucrose density centrifugation, numerous MVs with variable cargo, density and size can disperse over the gradient between 25% and 50% sucrose fractions [26]. While after centrifugation, assembled retroviruses would be detected in fractions between 36% and 41% of sucrose. Finally, it should be emphasized that membrane markers used to identify MVs (CD9, CD63, CD81, CD82, CD231, etc.) are not very specific. Indeed, these and the other markers might be present on the viral envelope as a part of the plasma membrane [36]. As noted above-the diameter and physical parameters of some MVs might be close to that of retroviruses. However, modified multi-step purification procedures, largely overwhelmed amounts of virus particles (minimally four orders of magnitude vs putative MVs content), lack of visible non-specific bands in SDS-PAGE, as well as the lack of proteasome subunits in identically non-infected HEK293T cells allow considering high purity of obtained preparations. It should be mentioned that our recent data on endogenous retroviruses [34] and data presented by others [37] indicate widespread hijack of virus elements or even virions during detachment of “large” MVs. However, such MVs, if loaded, can be successfully separated from the virus pool by filtration through the 0.22 μm membrane.

Previously, several groups revealed accumulation of 20S proteasome subunits as well as the 19S and PA28 proteasome regulators subunits inside viruses [14,18,20,21]. Thus, one cannot exclude that along with the 20S proteasomes, free proteasome regulators or even 26S proteasomes, or 20S proteasomes capped with PA28 complexes may be present in retroviral particles. Interestingly, ubiquitin is frequently detected inside virus particles [14].

**Figure 8:**
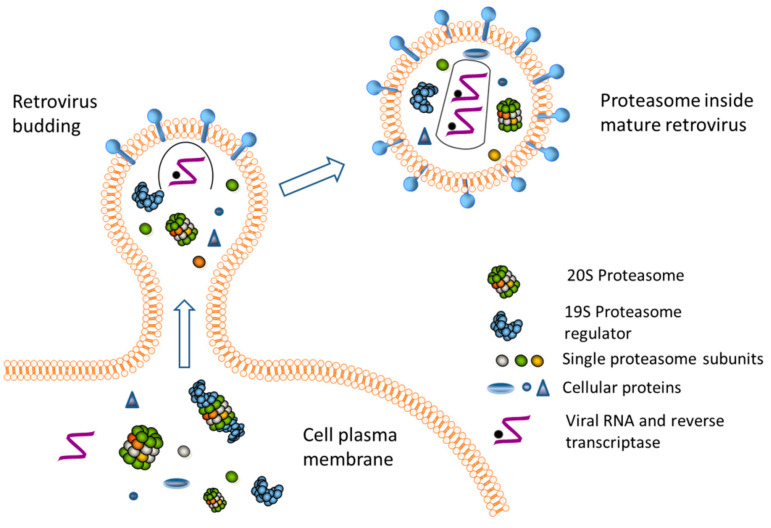
Putative mechanism of proteasome integration into retroviral particle. Proteasomes might occasionally be integrated into viral particles along with other cellular proteins and nucleic acids.

We consider that during the assembly of retroviruses, proteasomes might be trapped as cellular cargo (Figure 8). It is known that host cell proteins incorporate into virions and can serve certain functions [38,39]. Along these lines, Stegen et al. demonstrated that the incorporation of cellular proteins in the Herpes simplex virus is important for its proper propagation in cell culture [40]. In 1990-ies it was demonstrated that cyclophilin A interacts with HIV-1 Gag proteins, and it is essential for the capsid assembly. Disruption of cyclophilin incorporation can influence viral infectivity [41,42,43,44]. Although these data were recently argued [45], there are other examples highlighting the importance of the presence or absence of a particular host protein inside virions. For instance, uracil-DNA glycosylases or RNA-binding protein Staufen, the letter was suggested to play a role in viral RNA packaging [16]. Cellular proteins Ezrin and EHD4 were shown to be involved in the ability of Nef to increase virus infectivity [21]. On the other hand, HIV-1 Vif protein was shown to bind APOBEC3G to prevent its packaging into the virions [16]. Moreover, recent publications highlight tight crosstalk between viruses and ubiquitin-proteasome system, which is necessary for proper propagation of the virus and limitation of cellular defense capacity [46,47,48,49,50,51,52]. Thus, for instance, HIV-1 and HIV-2 are dependent on proteasome activity since proteasome inhibition was shown to interfere with Gag processing, release, and maturation [50].

Many cellular proteins were revealed as substrates of the 20S proteasomes [7,53], on the other hand, some of these proteins were previously indicated as components of the host-protein cargo of the HIV-1 [14] (Table 2). The half-life time of proteasome depends on the variant and temperature and might be from 1 to 5 days [54]. In contrast, the half-life of HIV-1 depends on conditions and may be nearly 48 h at 37 °C. Thus, it cannot be excluded that “during the trip” inside retroviruses, proteasomes might cleave the protein cargo, affecting the viral fitness and further propagation. This might have a bigger value if we consider that several viral proteins are degraded by proteasomes, including, for instance, the integrase [49], Vpr [55], Nef [56], Tat [57] (though Tat is not normally present within the HIV-1 virion). Hence, a question arises: Can proteasomes during transportation damage or completely neutralize retroviruses? If so, this may partially explain a rather high number of defective virus particles. It might also coincide with the probability of proteasome integration into the virion.

If, indeed, active proteasomes are present in retroviruses, it raises several additional questions:

1. Can the maturation of some retroviruses be influenced by proteasomes?
2. How frequently can functional proteasomes be detected in retroviruses?
3. What conditions for successful cleavage of proteins by proteasomes are required?

At the same time, the study has certain limitations. The precise localization of proteasomes within the viral particle remains unknown. Degradation of cellular and viral proteins by proteasomes within viral particles needs to be shown. Additionally, proteasome activity in viral preparations could be lower since certain contributions of the viral protease in fluorogenic substrate cleavage could not be ruled out. Further studies are necessary to clarify these issues and find out whether proteasome presence is “good”, “neutral”, or “bad” for the virus. In summary:

- Purification of retroviruses was optimized.
- Proteasome subunits and functional proteasomes are most likely trapped during the assembly of retroviruses.
- About 0.1–1 proteasome per virion might be present in retroviruses.
- 20S proteasome activity was detected in highly pure retroviral preparations.
- Outcome of the proteasome activity in retroviruses is not yet understood.

## 4. Materials and Methods

### 4.1. Cell Lines, Producing Cells and Viruses

Human embryonic kidney cell line HEK293T (ATCC CRL-3216) and HEK293T cells expressing EGFP were maintained in DMEM supplemented with 10% heat-inactivated fetal bovine serum (FBS), 1% penicillin-streptomycin and L-glutamine. In this study, HEK293T cells were also grown on the FBS-free cell culture medium Liforcell (Lifeblood Medical, Adelphia, NJ, USA). Cell lines were tested for mycoplasma contamination using a MycoSensor PCR assay kit (Agilent Technologies, Santa Clara, CA, USA) and were found mycoplasma-free.

Mouse mammary tumor virus (MMTV) was propagated in GR murine liver cells. Endogenous feline retrovirus (RD-114) was grown on human rhabdomyosarcoma RD-114 cells (ATCC CCL-136), endogenous Feline Leukemia Virus (FeLV) was propagated in Crandall-Rees feline kidney cells (ATCC, CCL94). Supernatants from GR liver cells, RD-114 cells and Crandall-Rees kidney cells were produced previously in NCI-Fredrick, Fredrick, MD, USA. Retroviruses mentioned above were double banded on sucrose gradients (provided by Prof. S. Oroszlan, NCI-Frederick, Frederick, MD, USA). An endogenous PERV-A expression clone was kindly provided by Dr. R Tonjes (Paul Echrich Institute, Langen, Germany). The JSRV expression clone was kindly provided by Prof. James DeMartini (Fort Collins, CO, USA). The pHIV-1_pNL4-3_ molecular clone (Catalog #114, AF3244930) was obtained from the NIH AIDS Research & Reference Reagent Program. Using site-directed mutagenesis (ThermoFisher Scientific, Waltham, MA, USA), two derivatives of pHIV-1_pNL4-3_ plasmids were prepared. The first expression plasmid was modified in SU by changing the REKR (3′-end) motive for the AEKA motive. The second expression vector was modified by elimination of the SU sequence, while the SU signal peptide sequence (MRVKEKYQHLWRWGWKWGTMLLGILMICSA) was cloned in pCR2.1 TOPO and after restriction ligated to 5′-end of TM.

Transfection of HEK293T cells was performed in six-well plates when cells were at 50% of confluence (5 × 10^5^ cells). Four μL of TransIT-293 transfection reagent (Mirus Bio LLC, Madison, WI, USA) per one μg of plasmid were applied. Supernatants were collected after 72 h and processed as described below.

To obtain fluorescent cells, the HEK293T cell line was transfected with a pEGFP-N1 (Takara Bio USA Inc., San Jose, CA, USA) expression vector. Transfection was performed with Mirus transfection agent (Mirus Bio LLC, Madison, WI, USA). The efficiency of transfection was controlled by fluorescent microscopy. After five passages (to confirm stable expression), transfected cells were cloned. Cells with the highest level of expression among the clones were split again. Finally, three cell lines with the best EGFP expression level were obtained, and one of them was used to study extracellular particles. Obtained cell lines stably expressed EGFP, presumably because of plasmid integration.

### 4.2. Sucrose Gradient Centrifugation

Cell supernatants were harvested on the third day after cell splitting. A total of 100, 200, or 250 mL of supernatant were collected and centrifuged twice at 1000× *g* and 3000× *g* for 12 min (each time) to eliminate cells and cellular debris, respectively. Obtained supernatants were filtered through 0.22 μm or 0.45 μm membranes (Millipore, Burlington, MA, USA). The supernatant was applied on 20% or 25% sucrose cushion in TN buffer (10 mM Tris-HCl, 150 mM NaCl, pH 7.6) and centrifuged at 120,000× *g* for 1 h 45 min at 4 °C. The pellet was dissolved in TN buffer to achieve the final 200–250-fold concentrates and applied on the sucrose density gradient (20–60% or 15–60%). Centrifugation was performed in 13 × 51 mm or 14 × 89 mm centrifuge tubes (Beckman Coulter, Palo Alto, CA, USA) at 160,000× *g* or 110,000× *g* for 4 h or 5 h at 4 °C. After centrifugation gradient fractions were diluted in TN buffer (1:5) and pelleted at 160,000× *g* for 1 h 30 min at 4 °C. Pellets were diluted in TN buffer and were kept frozen at −80 °C before use. Supernatant from HEK293T was processed as virus-containing supernatants and was used as a negative control.

### 4.3. Cellular Lysates

To prepare lysates, cells were washed four times with phosphate-buffered saline (PBS) or TN buffer and lysed in NP40 buffer (1% NP-40; 150 mM NaCl; 50 mM Tris-HCl pH 8.0) containing protease inhibitor cocktail Complete (Roche, Mannheim, Germany). After 10 min on ice, the lysates were centrifuged at 10,000× *g* for 10 min. The supernatant was recovered and used immediately or was kept frozen at −80 °C before use.

### 4.4. Primary and Secondary Antibodies

Primary mouse anti-20S proteasome α 1, 2, 3, 5, 6, 7 subunits monoclonal antibodies (Enzo, Farmingdale, NY, USA), mouse anti-20S proteasome β2 subunit monoclonal antibody (Enzo, Farmingdale, NY, USA), rabbit anti-20S proteasome β1 subunit polyclonal antibody (Abcam, Cambridge, UK) and rabbit anti-20S proteasome β5 subunit polyclonal antibody (GeneTex, Irvine, CA, USA) were used to detect proteasome subunits by Western blotting. Anti-p27 JSRV were kindly provided by Prof. DeMartini (Fort Collins, CO, USA), and Mab anti-gp41HIV-1 (2F5) was kindly provided by Dr. H. Katinger (Polymun, Vienna, Austria). Sheep anti-p24 CA HIV-1 and goat anti-gp120 HIV-1 were obtained from the Institute of Human Virology (Baltimore, MD, USA), and goat anti-p26 MMTV was obtained from NCI-Frederick (Frederick, MD, USA). Secondary antibodies that were used in this study: goat anti-mouse HRP-labeled antibodies (Enzo, Farmingdale, NY, USA), goat anti-rabbit HRP-labeled IgG (Abcam, Cambridge, UK), rabbit anti-goat IgG antibodies (Dako laboratories, Glostrup, Denmark). Development was performed using the ECL Prime kit (GE Healthcare Limited, Pollards Wood, UK). The ImageJ ver. 1.53o (US National Institutes of Health, Bethesda, MD, USA) software was used for the quantification of revealed protein bands.

### 4.5. Protein Electrophoresis and Western Blot Analysis

Electrophoresis of viruses and commercial 20S proteasomes (Enzo, Farmingdale, NY, USA) was performed using 4–20% gradient Tris-Glycine polyacrylamide gels (Serva, Heidelberg, Germany) and Tris-Glycine buffer (Novex, Life Technologies, Carlsbad, CA, USA). Gels were calibrated using PageRuler Plus pre-stained protein ladder and MagicMark XP Protein Standard (ThermoFisher Scientific, Waltham, MA, USA), or Prestained Protein Marker II (10–200 kDa) (Servicebio, Wuhan, China), or Western blot standard (Serva, Heidelberg, Germany). After electrophoresis, the proteins were transferred onto nitrocellulose membranes (Protran BA83, Whatman GmbH, Dassel, Germany) at 45 V for 2 h. To monitor transfer quality, the membranes were first stained with Ponceau S (Sigma-Aldrich, St. Louis, MO, USA). After distaining, the membranes were blocked with 6% dry milk in PBS-Tween for 2–3 h at room temperature. Incubation with primary antibodies was performed for 2–3 h at room temperature or overnight at 4 °C. After 5 times washing (5 min each) in PBS-Tween the membranes were incubated with secondary antibodies for 1 h at room temperature. The membranes were washed 5 times with PBS-Tween, treated with Pierce ECL Western blotting substrate (Pierce, Rockford, Tempe, AZ, USA) for 1 min, and exposed to Hyperfilm ECL (GE Healthcare Limited, Pollards Wood, UK).

### 4.6. Fluorescent and Transmission Electron Microscopy (TEM)

The HEK293T cells were seeded on poly-L-lysine coated glass-bottom µ-Dishes (Ibidi GmbH, Martinsreid, Germany). Images were captured using an LSM 780 fluorescent microscope (Carl Zeiss, Oberkochen, Germany) and a digital inverted fluorescent microscope EVOS FL (Thermo Fischer Scientific, Waltham, MA, USA)

Preparation of samples for thin sections (60–70 nm) was performed as described previously [58]. Virus-producing cells were fixed with 2.5% glutaraldehyde in HEPES buffer (pH 7.2), post-fixed in 1% osmium tetroxide, 0.1% tannic acid and 2% uranic acetate, and embedded in epon resin. Ultrathin sections of samples were examined using a JEM-2100 electron microscope (JEOL Csorp., Akishima, Japan) operated at 200 kV. Images were recorded with a CCD camera and 2048 × 2048 pixels.

### 4.7. Determination of Proteasome Activity 

Proteasome activity was measured using two approaches. First, we used Me_4_BodipyFL-Ahx_3_Leu_3_VS (UbiQbio, Amsterdam, The Netherlands) to estimate the proteasome activity using SDS-PAGE according to the protocol [59]. Shortly, serial dilutions (100, 50, 25, 12.5, 6.75, and 3.37 ng) of human 20S proteasome (Enzo, Farmingdale, NY, USA), 15 µL of JSRV (F4), 15 µL of HEK293T (F4) and 2.5 µL of FeLV were mixed with 1 µL of probe in PBS and incubated for 1 h at 37 °C. Samples were loaded into 15% Tris-Glycine polyacrylamide gel, and the fluorescence was analyzed at the excitation wavelength 480 nm and emission wavelength 530 nm using ChemiDoc XRS+ imaging system (Bio-Rad, Hercules, CA, USA). Then the gel was stained with ROTI Blue quick (Carl Roth, Karlsruhe, Germany).

The chymotrypsin-like and peptidyl-glutamyl peptide hydrolyzing (caspase-like) activities of proteasomes in viral preps were determined similarly as described [5]. In brief, two fluorogenic substrates, Suc-LLVY-AMC (Enzo, Farmingdale, NY, USA) and Z-LLE-AMC (Enzo, Farmingdale, NY, USA), were used to estimate corresponding activities. The tests were performed at least in duplicates. Viruses were disrupted by eight cycles of freezing/thawing. Aliquots (6 µL) of HEK293T, JSRV, HIV-1 AEKA fractions (F4) and 1 µL of RD-114, 5 µL of MMTV and 2 µL of FeLV were incubated in 100 μL of the reaction buffer (RB), containing 40 mM Tris-HCl (pH 7.5), 1 mM DTT, 5 mM MgCl_2_, 1 mM ATP, and 90 μM of substrate for 1 h at 37 °C. Six nanograms of purified human 20S proteasome (Enzo, Farmingdale, NY, USA) were used as a positive control. Control reactions with 100 nM of the proteasome inhibitor Bortezomib (Tocris, Bristol, UK) were performed to test nonspecific degradation of substrates. Reactions were stopped with 2% SDS solution (in ddH_2_O). Fluorescence at the excitation wavelength of 380 nm and emission wavelength of 440 nm was measured using a VersaFluor Fluorometer (Bio-Rad, Hercules, CA, USA). To calculate relative activity levels, the activity levels in probes with Bortezomib were subtracted from the values detected in samples containing viral preparations.

### 4.8. Software

The NCBI Blast software ver. 2.12.0 was used for the database search. Parameters of PCR primers were selected and analyzed using the OligoAnalyzer 3.1 (Integrated DNA Technologies, Coralville, IA, USA). Sequences were aligned using Lasergene Version 10 (DNASTAR, Inc., Madison, WI, USA). The image processing program ImageJ (US National Institutes of Health, Bethesda, MD, USA) was used to compare protein band intensity. The ProP 1.0 Server (https://services.healthtech.dtu.dk/, accessed on 17 January 2022) was used to predict furin cleavage sites.

## Figures and Tables

**Table 1:** Amount of CA in retroviruses and estimated viral content. * CA band intensity was determined using ImageJ software and data shown in Figure 5. CA total amount and quantity of viral capsids were estimated considering that a single virion contains 0.1 fg of CA proteins or ~1500–2000 molecules [25]. F4-fraction four of the gradient.

Purified Viruses	Load µL	CA Band Intensity *	Relative Band Intensity per 1 μL Load	CA Content µg/µL	Estimated Total CA Content	Viral Capsids per µL
pNLd F4	7	1959/7	279	0.024	4.8 × 10^11^	2.4 × 10^8^
pNLAEKA	7	2948/7	421	0.057	1.14 × 10^12^	5.7 × 10^8^
JSRV F4	7	5338/7	762	0.139	2.78 × 10^12^	1.39 × 10^9^
FeLV	1	8267	8267	1.67	3.34 × 10^13^	1.67 × 10^10^
RD-114	1	6452	6452	1.24	2.48 × 10^13^	1.24 × 10^10^
MMTV	5	3349/5	670	0.1	2 × 10^12^	10^9^
PERV F4	7	2625/7	375	0.047	9.4 × 10^11^	4.7 × 10^8^

**Table 2:** Cellular protein targets for functional 20S proteasome in retroviruses.

	Proteins (Protein Cargo of Retroviruses)	Function
**1**	Annexin A5	Blood clotting, interaction with apoptotic cells
**2**	Glyceraldehyde-3-phosphate dehydrogenase (GAPDH)	Metabolism, catalizes glycolysis, initiation of apaptosis, etc
**3**	Poly(rC)-binding protein 1	Multifunctional, nuclear export, cytosolic iron chaperon
**4**	6-phosphogluconate dehydrogenase	Metabolism, a key enzyme that produces NADPH
**5**	GDP dissociation inhibitor beta	Regulation of Rab targeting
**6**	Vimentin	Element of cytoskeleton, maintain cell integrity
**7**	Alpha-enolase	Multifunction, glycolitic enzyme
**8**	Hsp70	Protein folding, a member of chaperone family
**9**	Hsp60	Protein folding, a member of chaperone family
**10**	Ras GTPase-activating-like protein	Actin cytoskeleton, down regulate Ras activity

## Data Availability

The data is available upon request.
